# Perceived health competence and health education experience predict health promotion behaviors among rural older adults: A cross-sectional study

**DOI:** 10.1186/s12889-022-14080-1

**Published:** 2022-09-05

**Authors:** Xia Xie, Jiao Du, Jie He, Youchi Liu, Zihao Li

**Affiliations:** 1 Department of Nursing, Affiliated Hospital of North Sichuan Medical College, No.1 Maoyuan south road, Shunqing district, Nanchong, China; 2 School of Nursing, North Sichuan Medical College, No.55 Dongshun road, Gaoping District, Nanchong, China

**Keywords:** Health behaviors, Health promotion, Perceived health competence, Health education activities experience

## Abstract

**Background:**

Health promotion behaviors are key determinant of health and well-being, and also play an important role in promoting successful aging. This study investigated levels of engagement in health promotion behaviors among Chinese rural older adults, and explored effects of perceived health competence, health education activities experience and sociodemographic variables on health promotion behavior in this population.

**Methods:**

A multicenter cross-sectional survey was performed. Participants were recruited by a multistage, stratified, cluster-sampling procedure from Nanbu County, Sichuan Province, China. All participants completed four paper questionnaires: sociodemographic characteristics and health care status survey, the Chinese version of the health promoting lifestyle profile-II (HPLP-II), perceived health competence scale and Lubben social network scale. Data were collected from July to August 2021. Stepwise multiple linear regression analysis was performed to analyze the effects of different factors on health promotion behaviors.

**Results:**

A total of 425 rural older adults with an average age of 72.7 years were included in analysis. The overall average score of HPLP-II was 101.6. The stepwise multiple linear regression analysis results showed that those who had higher perceived health competence (*β* = 0.66, *P* < 0.001), experienced health education activities (*β* = 0.254, *P* < 0.001), had physical examination (*β* = 0.107, *P* < 0.001), was married (*β* = 0.189, *P* < 0.001), had primary school education or above (*β* = 0.189, *P* < 0.001), and had a per capita monthly household income of more than 1000¥ (*β* = 0.085, *P* = 0.007), have higher levels of engagement in health promotion behaviors; while the level of health promotion behaviors of the older adults living alone was lower than that of living with their spouse or others (*β* = -0.192, *P* < 0.001). Combination of the above variables accounted for a total of 69.1% of the variance in health promotion behaviors. *Conclusions*: The level of health promotion behaviors among Chinese rural older adults is low. Perceived health competence and health education activities experience are two strong determinants of health promotion behaviors. Comprehensive health promotion programs aimed at improving perceived health competences and health literacy through health education activities may be an important part of optimizing the level of health promotion behaviors among rural older adults.

## Background

China is one of the fastest ageing countries in the world, with the largest absolute number of older adults. It is reported that the number of people aged 60 and over in China has reached 264 million in 2020, accounting for 18.29% of the total population [1]. Previous studies have shown that 61.36% of Chinese older adults live in rural areas where the economic situation is poor [2]. The aging of the population triggers a series of problems, such as declining physical function, and increasing prevalence of chronic diseases and psychological problems [3, 4]. Therefore, finding effective ways to guarantee and improve the health level of rural older adults has become an important challenge to be addressed urgently. Health promotion behaviors have been identified as a key determinant of health and well-being, and also play an important role in promoting successful aging [5, 6]. Identifying the level of health promotion behaviors among older adults and exploring its key predictors are prerequisites for developing effective health promotion behaviors interventions among older adults.

Many previous studies have been conducted to investigate the level of health promotion behaviors among older adults [5, 7–10]. However, most of them focused on urban older adults [7, 8], or analyzed urban and rural residents as a group, without taking into account differences in where they lived [5, 9, 10]. Several studies have shown that the level of health promotion behaviors among older adults is affected by the living area [5, 9, 10]. Therefore, separate studies should be conducted to investigate the level and key predictors of health promotion behaviors in rural or urban older adults, thus providing references for the development of targeted interventions.

In previous studies, several factors have been explored as potential predictors of health promotion behavior. The main factors involved included gender [5, 10, 11], age [10, 12], living area [5, 9, 10], education status [5, 10, 12, 13], income [13], marital status [5, 10], regular physical examination [12, 13], social network [12, 14] and general self-efficacy [7, 12, 15, 16]. The relationships between most of the above factors and health promotion behavior in different studies are inconsistent, and further studies are needed.

Perceived health competence is the degree to which an individual feels capable of effectively managing his or her health behaviors and health outcomes [17]. According to the knowledge, attitude, belief and practice model, knowledge and attitudes/beliefs are key variables for behavior change [18]. Based on this model, older adults with health education experience are likely to have higher levels of health promotion knowledge and perceived health competence, and thus have higher levels of health promotion behaviors. However, few studies have focused on the relationship between perceived health competence, health education experience, and health promotion behaviors among rural older adults.

This study was conducted to investigate the levels of health promotion behavior among Chinese rural older adults, and to explore effects of perceived health competence, health education activities experience and sociodemographic variables on health promotion behaviors.

## Methods

### Study design and participants

This study was a multicenter cross-sectional survey conducted in Nanbu County, Sichuan Province, China. Rural community-dwelling residents who: were aged 60 years or older; resided in selected villages; had lived there for at least 1 year preceding the survey date; were able to understand and respond to the questions on the questionnaire were invited to participate in the study. Those who lived in selected villages but were housed in nursing homes or were unwilling to participant in the study were excluded.

### Sampling

We used a multistage, stratified, cluster-sampling procedure, which considered geographical region and economic development status. In stage 1, Nanbu County, Sichuan Province, China was selected. Sichuan province can be classified as economically developed area in western China and plays an important role in the overall development of the country. Nanbu County, located in the northeast of Sichuan Province, is a typical area with serious aging of the older population, higher than the national and Sichuan average (Table 1), and the aging phenomenon of rural population in Nanbu Country is more prominent than that of urban population. Therefore, Nanbu Country, Sichuan Province was selected as the sample region. The social-economic characteristics of the sample region are shown in Table 1. Nanbu County has 38 townships and 363 administrative villages, each village has nearly 10 village groups and about 200 households. In stage 2, five townships were randomly selected from 38 townships. In stage 3, one administrative village was randomly selected from each selected township. In sage 4, three village groups were randomly selected from each selected administrative village. Finally, we screened all eligible individuals in the 15 village groups as the sample population for this study.

**Table 1:** Social-economic characteristics of sample region in 2020

Region	Population (10,000)	Population aged over 60 (10,000)/(%)	Rural per capita disposable income (¥)
Nanbu Country^a^	81.72	23.00(28.14)	18,083
Sichuan Province^b^	8,367.49	1,816.38(21.71)	15,929
China^c^	144,349.74	26,401.88(18.29)	17,131

^a^The data sources come from the offcial website: https://www.scnanbu.gov.cn/show/2021/07/09/37945.html and https://www.scnanbu.gov.cn/show/2021/06/28/37675.html

^b^The data sources come from the Sichuan Provincial Bureau of Statistics: http://tjj.sc.gov.cn/scstjj/tjgb/common_list.shtml and http://tjj.sc.gov.cn/scstjj/tjgb/2021/3/14/c64ac94ca86c4714adaf117789e47073.shtml

^c^The data sources come from the National Bureau of Statistics of the People’s Republic of China: http://www.stats.gov.cn/tjsj/tjgb/rkpcgb/ and http://www.stats.gov.cn/tjsj/zxfb/202102/t20210227_1814154.html

### Measures

All participants completed four paper questionnaires: sociodemographic characteristics and health care status survey, the Chinese version of the health promoting lifestyle profile-II (HPLP-II), perceived health competence scale (PHC) and Lubben social network scale.

### Sociodemographic characteristics and health care status survey

Sociodemographic characteristics including gender, age, education status, marital status, per capita monthly household income, proportion of living with alone, smoking and drinking, were investigated. The questionnaire also addressed the following: regular physical examination and health education activities experience. These indicators were measured by the following questions: Have you had a regular physical examination during the past year? Have you participated in any health education activities before this survey? For each question, the response options included “yes” and “no”.

### The Chinese version of the health promoting lifestyle profile-II (C-HPLP-II)

The health promoting lifestyle profile-II developed by Walker [19], translated and validated by Cao [20], was used to assess health promotion behaviors. The C-HPLP-II consists of 6 dimensions with a total of 40 items: interpersonal relationship (5 items), responsibility for health (11 items), stress management (5 items), diet (6 items), physical activity (8 items), spiritual growth (5 items). Each item is rated on a four-point Likert scale with a range of 1 (not at all) to 4 (always). The mean score was calculated with a higher score indicating higher levels of engagement in health promotion behaviors. The original scale had a Cronbach’s α coefficient of 0.94 at the time of its development. The Cronbach’s α coefficient of the scale in this study was 0.907.

### The Chinese version of the perceived health competence scale (C-PHC)

The perceived health competence scale (PHC) by Smith [17], translated and validated by Liang [12], was used to assess perceived health competence. The C-PHC consists of 8 items on a five-point Likert scale. Respondents were asked how much they agreed with each item with a range of 1 (do not agree at all) to 5 (absolutely agree). The mean score was calculated with a higher score indicating higher perceived health competence. The original scale had a Cronbach’s α coefficient of 0.90 at the time of its development. The Cronbach’s α coefficient of the scale in this study was 0.893.

### The Chinese version of the Lubben social network scale (C-LSNS)

The Lubben social network scale (LSNS) by Lubben [21], translated and validated by Qi [22], was used to assess the credible relationships among older adults with family/relatives and friends and the support they can get from them. The C-LSNS consists of 12 items. The score for each item ranges from 0 to 5, with a total score of 0–60. The higher the score, the richer the respondent's social network. The Cronbach’s α coefficient of the scale in this study was 0.792.

### Data collection procedures

For data collection, the researchers informed the heads of selected village groups of the purpose of this study and obtained permission to conduct research in these places. In this study, 3 researchers and 9 research assistants formed three survey teams. Each team, including a researcher and three assistants, was in charge of the collection of data from a village group. All research assistants were senior nursing students. For inter-rater reliability, the survey teams were trained by the primary investigator on the contents of the questionnaire and survey techniques. The investigators then visited selected village groups and identify potential participants who were interested in participation by conducting household visits. They were screened for eligibility to participate, and if they were eligible to participate, the purpose and procedures of the study were explained to them. After written consent was obtained, a face-to-face interview was conducted using structured questionnaires. Participants completed the questionnaire themselves with a pen if they were able to do so. The completed questionnaires were then reviewed and collected by surveyors. For participants with literacy difficulties, mobility problems, or poor vision, a surveyor read the questions aloud and recorded the participants' responses to the questions. Data were collected from July to August 2021. A total of about 700 older adults were visited, of whom 273 were excluded from the study because of severe cognitive or communication disabilities (*n* = 81), unwillingness to participate in this study (*n* = 186), or local residence of less than one year (*n* = 6). The remaining 427 older adults who met the inclusion criteria were investigated. During the survey, two older adults dropped out and failed to complete the questionnaire. Therefore, data from the remaining 425 older adults were included in the final analysis.

### Data analysis

Descriptive statistics, including number, percentage, mean and standard deviation, were used to summarize the characteristics of participants and the levels of health promotion behaviors. Differences in health promotion behaviors according to sociodemographic characteristics and health care status were analyzed using t-tests. Pearson’s correlation coefficients were calculated to determine associations of health promotion behaviors with age, perceive health competence and social network. Stepwise multiple linear regression analysis was performed to analyze the effects of different factors on health promotion behaviors. All statistical analyses were performed using IBM SPSS version 25, and a *P*-value less than 0.05 was considered statistically significant.

## Results

### Characteristics of the participants

Table 2 shows the characteristics of participants. The mean age of participants was 72.7 ± 7.0 years and most of them were women (71.8%). Less than half of participants were single (40%) and lived alone (37.6%), while the others lived with a spouse, adult/child or both. The majority of participants reported a low education level with 64.7% having no education. Most participants (78.8%) had a per capita monthly household income < 1000¥, but more than four-fifths of them had a regular physical examination during the past year. A total of 68.2% participants had no any experience of health education activities. The mean PHC and LSNS scores was 26.4 and 23.6, respectively, indicating poor perceived health competence and social network of this group of population.

**Table 2:** Characteristics of participants (*N* = 425)

Variables	Categories	n( %) or M ± SD
Gender	Male	120(28.2)
	Female	305(71.8)
Age		72.7 ± 7.0
Education status	No education	275(64.7)
	Elementary school and over	150(35.3)
Marital status	Single^a^	170(40.0)
	Married	255(60.0)
Living with alone	Yes	160(37.6)
	No	265(62.4)
Per capita monthly household income (¥)	< 1000	335(78.8)
	≥ 1000	90(21.2)
Smoking	No	360(84.7)
	Yes	65(15.3)
Drinking	No	305(71.8)
	Yes	120(28.2)
Regular physical examination	No	65(15.3)
	Yes	360(84.7)
Health education experience	No	290(68.2)
	Yes	135(31.8)
Perceived health competence		26.4 ± 4.8
Social network		23.6 ± 9.7

*Note. M* Mean, *SD* Standard deviation; a: unmarried, divorced, widowed

### Level of health promotion behaviors of participants

The overall average score of HPLP-II was 101.6 ± 12.9. The average item score for each of the six dimensions of health promotion behaviors were 2.85 in interpersonal relationship, 2.71 for nutrition, 2.70 for stress management, 2.65 for spiritual growth, 2.57 for physical activity, and 2.16 for responsibility for health.

### Differences in health promotion behaviors according to sociodemographic characteristics and health care status

As shown in Table 3, significant differences in participants’ levels of engagement in health promotion behaviors were found according to most of the sociodemographic and health care variables, excluding smoking.

**Table 3:** Differences in health promotion behaviors according to sociodemographic characteristics and health care status (*N* = 425)

Variables	Categories	M ± SD	*t*
Gender	Male	105.7 ± 11.7	4.346^***^
	Female	100.0 ± 13.1	
Education status	No education	97.8 ± 12.7	-9.301^***^
	Elementary school and over	108.5 ± 10.5	
Marital status	Sigle^a^	98.9 ± 14.0	-3.368^**^
	Married	103.4 ± 12.0	
Living with alone	No	103.7 ± 12.3	4.359^***^
	Yes	98.1 ± 13.4	
Per capita monthly household income (¥)	< 1000	101.0 ± 13.6	-2.158^*^
	≥ 1000	103.8 ± 10.4	
Smoking	No	101.2 ± 13.2	-1.752
	Yes	104.0 ± 11.8	
Drinking	No	100.7 ± 13.9	-2.49^*^
	Yes	103.8 ± 10.0	
Regular physical examination	No	94.6 ± 10.6	-5.563^***^
	Yes	102.9 ± 13.0	
Health education experience	No	97.6 ± 11.5	-10.306^***^
	Yes	110.1 ± 12.0	

*Note. M* Mean, *SD* Standard deviation; a: unmarried, divorced, widowed

^*^P < 0.05

^**^P < 0.01

^***^P < 0.001

### Correlations of health promotion behaviors with age, perceive health competence and social network

Perceived health competence (r = 0.724, *P* < 0.001) and social network (*r* = 0.184, *P* < 0.001) was significantly positively correlated, age (*r* = 0.184, *P* < 0.001) was significantly negatively correlated with health promotion behaviors.

### Results of stepwise multiple linear regression analysis

The stepwise multiple linear regression analysis results showed that those who had higher perceived health competence, experienced health education activities, had physical examination, was married, had primary school education or above, and had a per capita monthly household income of more than 1000¥, had higher levels of engagement in health promotion behaviors; while the levels of health promotion behaviors of the older adults living alone was lower than that of living with their spouse or others (Table 4). Combination of the above variables accounted for a total of 69.1% of the variance in health promotion behaviors, F (417) = 133.245, *P* < 0.001, with an adjusted R^2^ of 0.686.

**Table 4:** Results of stepwise multiple linear regression analysis (*N* = 425)

Independent variables	*β*	*P*	95% CI
Perceived health competence	0.66	< 0.001	1.637, 1.954
Health education experience	0.254	< 0.001	5.436, 8.716
Education status	0.189	< 0.001	3.457, 6.82
Per capita monthly household income	0.085	0.007	0.732, 4.695
Regular physical examination	0.107	< 0.001	1.78, 5.948
Living with alone	-0.192	< 0.001	-7.39, -2.874
Marital status	0.189	< 0.001	2.677, 7.35

*Note. CI* Confidence interval. R^2^ = 0.691(69.1%); adjusted R^2^ = 0.686 (68.6%), F(417) = 133.245, *P* < 0.001

## Discussion

In this study, C-HPLP-II was used to investigate levels of engagement in health promotion behaviors among rural older adults in Nanbu County, Sichuan Province, China. The result showed that the overall average score of HPLP-II was 101.6, accounting for only 63.1% of the total score of the scale. It indicates that the overall levels of health promotion behaviors of this group of population was relatively low. It is necessary to develop and implement programs that encourage the rural older persons to engage in health promotion behaviors.

The average item score for each of the six dimensions of the C-HPLP-II was also calculated in this study. The dimensions that scored highest were interpersonal relationship and nutrition, and lowest were responsibility for health. The results are similar to those of studies in other countries or regions [10, 13]. This phenomenon may be explained by the fact that Chinese people have always attached importance to the maintenance of interpersonal relationships [23], and with the development of rural economy, rural older adults gradually pay attention to dietary ingredients and nutritional management [24]. The lowest score on health responsibility dimension may be related to the fact that few rural older adults are aware of their responsibility to be proactive in maintaining their health. Previous studies have provided evidence that older adults are not free from age-related stereotypes when they are faced with health problems [25]. For example, older adults tend to believe that the decline in their physical and cognitive functions is a natural aging process, so health care is unnecessary [26]. Additionally, they often express negative beliefs about the curability and controllability of health problems [27]. This low expectation of ageing may also be related to the inactive involvement of older adults in taking responsibility for their own health [28]. Therefore, interventions focused on promoting active ageing is recommended.

Stepwise multiple linear regression analysis was performed to examine the amount of variance in health promotion behaviors explained by the independent variables (Table 4). The results showed the combination of perceived health competence, health education activities experience, education status, per capita monthly household income, regular physical examination, living with alone and marital status accounted for a total of 69.1% of the variance in health promotion behaviors. Of them, perceived health competence (*β* = 0.66, *P* < 0.001) and health education activities experience (*β* = 0.254, *P* < 0.001) had the greatest effect on the health promotion behaviors of older adults.

In the study, participants with higher levels of perceived health competence were found to have higher levels of engagement in health promotion behaviors. Perceived health competence is the individual's perception or efficacy of health [17]. Empirical evidence has shown that self-efficacy is associated with many positive outcomes, particularly in the area of health behavior [7, 12, 15, 16]. The study supports this connection by demonstrating that perceived health competence positively predict health promotion behaviors. In other words, this study further confirmed the potentially powerful role of health self-efficacy in improving health promotion behaviors among rural older adults. This is a reminder that health care providers may be able to improve the level of health promotion behaviors of rural older adults by making efforts to enhance the perceived health competence.

Another key finding of the study was that older adults who participated in health education activities had higher levels of health promotion behaviors than those who did not. The finding provide evidence that the levels of health promotion behaviors may be improved through health education campaigns. One possible explanation for the finding is that older adults who experienced health education activities have higher health literacy [29, 30]. Previous studies have confirmed that individuals with higher health literacy have higher levels of health behaviors [31, 32]. Given the high proportion (68.2%) of rural older adults who did not participant any health education activities in this study, it is urgent to carry out health education activities in this population to improve their levels of health promotion behaviors.

In addition, this study confirmed that education level, per capita monthly household income, regular physical examination and marriage positively predicted health promotion behaviors, while living alone was a negative predictor of health promotion behaviors. These findings are consistent with previous empirical evidence [5, 10, 12, 13]. It is suggested that these five sociodemographic and health care variables should be taken into account when formulating policies and prevention plans for health promotion behavior of rural older adults.

Notably, although several studies have shown associations of social networks, smoking, drinking and health promotion behavior in different groups [33–35], this association has not been confirmed in this study. One possible explanation is that the social networks of rural Chinese older people are mainly composed of local rural relatives and neighbors [36]. Rural people generally have lower levels of education and health promotion behaviors, as confirmed by this study. Another explanation is that the prevalence of smoking and drinking among rural women in China is very low [37, 38], and the majority of the study population in this study were women. Further studies on relationships between these variables and health promotion behaviors of rural older adults are warranted in other countries or regions.

The study has several limitations. First, the cross-sectional design of the study allows only for correlation, not causation. To solve this problem, longitudinal studies will be necessary in the future. Second, participants were recruited from only one county, which limited the generalizability of the study findings. However, given the similarities between the participants in the study and the census demographic statistics of older adults in Sichuan and China, the study is likely to be representative of older adults in other geographic regions of China. Moreover, environmental factors that hinder the maintenance of positive health promotion behaviors in rural settings are prevalent in many villages around the world, which may enhance the generalizability of this study in other regions of China as well as in rural areas of the world.

Despite these limitations, the study has several strengths. First, the study investigated levels of health promotion behaviors of a large number of Chinese rural older adults. Second, the effects of sociodemographic and health care characteristics, perceived health competence and social network on the health promotion behaviors of rural older adults were analyzed in this study. Perceived health competence and health education activities experience were found to have the greatest effect on health promotion behaviors. The results of the study may be used as basic data for the development of health promotion programs for Chinese rural older adults. At the same time, these findings have implications for the development of preventive policies and programs to promote health behaviors in other countries with a similar socioeconomic structures and cultural expectations for health behaviors.

## Conclusions

In conclusion, the level of health promotion behaviors among Chinese rural older adults is low. Perceived health competence and health education activities experience are the main predictors of health promotion behaviors in this group of population. It indicates comprehensive health promotion programs aimed at improving perceived health competences and health literacy through health education activities may be an important part of optimizing the level of health promotion behaviors among rural older adults. These findings have important implications for improving health promotion behaviors among older adults, thereby improving their health and promoting successful ageing in China and other countries or regions around the world that are experiencing demographic transition.

## Data Availability

The datasets used and/or analyzed during the current study are available from the corresponding author on reasonable request.

## Abbreviations

HPLP-II: Health promoting lifestyle profile-II
PHC: Perceived health competence scale
LSNS: Lubben social network scale
M: Mean
SD: Standard deviation
CI: Confidence interval
